# A Novel Single Nucleotide T980C Polymorphism in the Human *Carboxypeptidase E* Gene Results in Loss of Neuroprotective Function

**DOI:** 10.1371/journal.pone.0170169

**Published:** 2017-01-23

**Authors:** Lin Cong, Yong Cheng, Niamh X. Cawley, Saravana R. K. Murthy, Y. Peng Loh

**Affiliations:** 1 Department of General Surgery, Peking Union Medical College Hospital, Chinese Academy of Medical Sciences and Peking Union Medical College, Beijing, China; 2 Section on Cellular Neurobiology, *Eunice Kennedy Shriver* National Institute of Child Health and Human Development, National Institutes of Health, Bethesda, MD, United States of America; University of Louisville, UNITED STATES

## Abstract

Report of a human with a homozygous truncating null mutation of the *Carboxypeptidase E (CPE)* gene with endocrinological and neurological deficits prompted us to search for other mutations in the human *CPE* gene that might be linked to disease. We searched an EST database and identified from a small population of patients, a novel T to C single nucleotide polymorphism (SNP) in the *CPE* gene at bp980 of exon 4, herein called *TC-CPE*. This introduces a tryptophan to arginine (W235R) mutation in the catalytic domain of human CPE protein. Over-expression of TC-CPE in N2A cells, a neuroendocrine cell line, showed that it was synthesized, but was found in lesser amounts compared to over-expressed WT-CPE in these cells. Furthermore, TC-CPE was secreted poorly from these N2A cells. The levels of TC-CPE were significantly increased after the N2A cells were treated with MG132 (a proteasome inhibitor), suggesting that TC-CPE was targeted to proteasomes for degradation in N2A cells. In addition, TC-CPE induced ER stress as demonstrated by the increased expression of CHOP in N2A cells. Double labeling of CPE and calnexin (and ER marker) suggested the accumulation of TC-CPE in the ER, and the accumulation appears to be enhanced by the treatment of MG132 in the cells. Moreover, the secreted levels of TC-CPE were not affected by the treatment of MG132 in the cells. Over-expression studies revealed that while N2A cells transfected with WT-CPE showed reduced cytotoxicity when challenged with H_2_O_2_ compared to cells expressing an empty vector, cells transfected with TC-CPE had no effect. Furthermore, WT-CPE condition medium showed protective effect against oxidative stress, but not TC-CPE condition medium. Although co-expression of WT-CPE and TC-CPE in N2A cells resulted in the reduction in secretion of WT-CPE, co-expression of WT-CPE and TC-CPE did not significantly affect the protective effect of WT-CPE. Taken together, we have identified a novel SNP in the CPE gene which results in the loss of its neuroprotective function in cells and may confer neurological disorders in humans.

## Introduction

Genetic mutations lead to human diseases including endocrinological and neurological disorders. Newly described human gene mutations have been accumulating at a rate of about 10,000 every year, with approximately 300 new ‘‘inherited disease genes” being identified annually[1]. Examples of genetic diseases include Alzheimer’s disease (AD), a devastating neurodegenerative disease with the majority of cases manifesting as a late onset sporadic form[2]. Efforts to find the genetic factors contributing to AD have led to the discovery of *Amyloid precursorprotein (APP)*, *Presenilin 1 (PSEN1)*, and *Presenilin 2 (PSEN2)* as a cause of autosomal dominant AD, and ε4 allele of *Apolipoprotein E* (*APOE*) has been identified as a strong genetic risk factor for AD[3], in addition to at least 21 additional genetic risk loci[3]. Another example is obesity, a major clinical and public health concern with over 600 million adults affected globally as of 2014 (World Health Organization, 2014). Obesity is also associated with other diseases such as type 2 diabetes mellitus, cancer, fatty liver disease, hypertension and cardiovascular diseases [4]. Although the etiology of obesity is not fully understood, considerable evidence suggest that 40 to 70% of body mass index (BMI) variation is due to genetic factors[5]. Recently, 119 common gene variants have been identified for polygenic obesity traits in humans through GWAS[6]. The importance of genetic mutations in human diseases and its potential usage as therapeutic targets have driven a greater effort to search for new mutations in genes with physiological functions.

The *carboxypeptidase E (CPE)* is an obesity susceptibility gene[7] encoding a prohormone processing enzyme (initially described by Fricker LD[8] and HookVY [9] in 1982). The physiological functions of CPE have been found in both the endocrine and nervous systems[10]. CPE has been demonstrated to be a neurotrophic factor working extracellularly to protect neurons against various stressors through ERK and AKT signaling pathways[11]. The neuroprotective property of CPE is independent of its enzymatic activity and is similar to a classic neurotrophic factor such as BDNF. Therefore, CPE was given an alternative name, neurotrophic factor-alpha 1 (NF-α1), when referring to its extracellular functions [11]. The neuroprotective role of CPE is also supported by the *in vivo* study showing that mice lacking CPE expression exhibited neurodegeneration in the hippocampal CA3 region and learning and memory deficits[12]. Extracellular CPE was further found to be an endogenous anti-depressant agent [13] and involved in neural development and stem cell differentiation[14–16]. In addition, CPE is relevant in AD as a study showed aberrant CPE accumulation in brains from patients with AD[17]. CPE knock-out mice and mice bearing a Ser202Pro mutation led to endocrine and neurological disorders including obesity, diabetes, neurodegeneration and infertility [7,10,18], while a human CPE truncating null mutation found in a patient, exhibited obesity, type 2 diabetes and intellectual disability [19]. The numerous functions of CPE, its association with disease and the detrimental effect of the lack of CPE in humans and mice due to gene mutations prompted us to search for human CPE mutations that may be relevant to human diseases.

In this study we investigated the effects of a novel mutation in the *CPE* gene that was discovered through single nucleotide polymorphism database (dbSNP) Blast analysis. This mutation consists of a T to C SNP at bp980 of exon 4, which results in Tryptophan (W) to Arginine (R) substitution at codon 235 (W235R). The mutation is located in the catalytic domain of the enzyme and found in 12.5% of the AGI_ASP population of patients that were in their 20s when their blood was analyzed. The AGI_ASP population is made up of 40 African-Americans and Caucasians (dbSNPrs cluster id: rs34516004). Through *in vitro* cell biological studies, we show that the SNP caused loss of enzymatic activity in the CPE protein. It was retained in the endoplasmic reticulum (ER), degraded by proteasomes and poorly secreted compared to the WT-CPE. Furthermore, the CPE mutant was able to “hijack” the WT-CPE into the degradation pathway. Cell viability studies showed TC-CPE did not have neuroprotective function compared to WT-CPE. Thus, our present study identified a new SNP in the human *CPE* gene which leads to loss of its function in neuroprotection.

## Materials and Methods

### DNA constructs

The Open Reading Frame (ORF) of human *WT-CP*E and the *TC-CPE*mutant were chemically synthesized(GenScript, Piscatawy, NJ) and sub-cloned into the *pcDNA3*.*1(+)* plasmid with BamH1 and XhoI restriction sites to generate *WT-CPE/ pcDNA3*.*1* and *TC-CPE/ pcDNA3*.*1* expression vectors. Both of the constructs were sequenced to confirm their orientation and structure. The *pcDNA3*.*1* vector alone was used as an empty vector (EV) control.

### Cell culture and transfection

N2A cells and COS-7 cells were maintained in DMEM medium supplemented with 10% fetal bovine serum and pen-strep antibiotics (complete medium) and incubated at 37°C with 5% CO_2_. Once 80% confluency was reached, the cells were washed with Hank’s Balanced Salt Solution (HBSS) and transfected with the plasmids. Transfection reactions were conducted with the Lipofectine 3000 reagent kit according to the manufacturer’s instruction (Invitrogen, Carlsbad, CA). For the proteasome inhibition study, N2A cells were transfected with EV, WT-CPE and the TC-CPE mutantfor 24 h, then treatedfor a further 24 h with 5 μM MG132 (Sigma, St. Louis, MO) or vehicle(DMSO) before samples were collected for Western blot analysis.

### Protein extraction

Lysis buffer was prepared with T-PER lysis buffer (Thermo scientific, Waltham, MA) supplemented with 0.5% TritonX-100, a protease inhibitor cocktail (Roche, Indianapolis,IN)and PMSF (1mM). This buffer was kept ice cold.The media was aspirated and the cells washed twice with ice cold HBSS. One hundred μl of lysis buffer was added to each plate. The cells were harvested by scraping and transferred to 1.5ml Eppendorf tubes and kept on ice for 10 min. The samples were then centrifuged at 13000 rpm for 20 minand the supernatant transferred to a new set of tubes and stored at -20°C.

### Protein quantification

Protein lysates were quantified bythe BCA Protein AssayReagent (Thermo Fisher Scentific, IL) and readings were taken at 562nm in a spectrophotometer. The concentration of the protein samples were calculated based on a standard curve of BSA in solution.

### Western blot

Samples (30 μg total protein per lane) were separated by electrophoresis on a 12% sodium dodecyl sulfate-tris glycine gels (Invitrogen, Carlsbad CA). After transfer to nitrocellulose the membranes were blocked with 5% non-fat milk for 1 hour at room temperature and incubated in primary antibodies overnight at 4°C. The membranes were subsequently washed with Phosphate Buffered Saline-Tween 20 (0.1%) (PBS-T) three times and incubated with fluorescent conjugated anti-mouse or anti-goat secondary antibodies (LI-COR Biotechnology, Lincoln, NE) in darkness at room temperature for 1 hour. The membranes were scanned by the Odyssey infrared imaging systems version 2.1 (LI-COR Biotechnology, Lincoln, NE) and the bands were quantified by the Odyssey software. Primary antibodies used were mouse CPE monoclonal antibody (R&D Systems, Inc, Minneapolis, MN) at 1:2000, monoclonal mouse anti-CHOP antibody at 1:2000 (Cell Signaling, Danvers, MA), mouse beta-actin monoclonal antibody (1:5000, Cell Signaling).

### Protein secretion analysis

N2A cells were transfected with EV, WT-CPE and TC-CPE. After incubation of the cells for 24 hours at 37°C, the medium was removed and substituted with DMEM medium with 0.01% FBS. Twenty four h later the medium was collected. Western blot analysiswas performed on the secreted proteins as described above with the exception that 26μl of the medium was used.

### High pressure liquid chromatography (HPLC)

To determine CPE enzymatic activity, adrenocorticotropin (ACTH)1-17 was used as a substrate. This peptide has a lysine-lysine-arginine amino acid sequence at its carboxyl-terminus. CPE cleaves these basic amino acids sequentially from the ACTH(1–17) to generate ACTH(1–16), ACTH(1–15) and ACTH(1–14), which can be identified by HPLC. Two μg of ACTH(1–17) were incubated in 50 mM sodium acetate, pH 5.0 for 18 h with conditioned media from COS-7 cells expressing either WT or mutant CPE or with media from non-transfected COS-7 cells. In a duplicate set of tubes, the reactions were carried out in the presence of 2mM cobalt chloride. Cobalt stimulates the enzymatic activity of CPE by displacing the zinc that is normally found in the enzyme’s active site and catalyzing the reaction more efficiently. A third set of tubes were set up that were identical to the second set, but also contained 25μM GEMSA, a potent specific inhibitor of CPE. All enzyme reaction products were analyzed by HPLC. The HPLC system used buffer A (0.1% trifluoroacetic acid (TFA)) and buffer B (80% acetonitrile/0.1% TFA). The column (Jupiter; C18-reverse phase column) was equilibrated in 30% buffer B and the gradient went to 34% buffer B over 12 minutes. In this case, ACTH(1–17) elutes first at ~5 min followed closely by ACTH(1–16) and then by ACTH(1–15) and ACTH(1–14). The peptides were monitoredby absorbance at 214 nm.

### Quantitative RT-PCR

Quantitative RT-PCR was used to analyze the transcript expression of CPE after transfection of EV, WT-CPE and TC-CPE in N2A cells. Quantitative RT-PCR was performed as described previously[11]. Primer sequences for CPE-fwd: 5’-CTCATCAGCTACCTGGAGCA-3, rev:5’-AGCAAGCAATCGCCAGTAAT-3’; for 18S-fwd: 5′-CTCTTAGCTGAGTGTCCCGC-3′, rev: 5′-CTGATCGTCTTCGAACCTCC-3′.

### Lactate dehydrogenase (LDH) release assay

LDH release assay was used to measure the cytotoxicity of N2A cells after transfection of EV, WT-CPE and TC-CPE in the presence or absence of H_2_O_2_. This was achieved with a CytoTox 96 Non-Radioactive Cytotoxicity Assay kit following the manufacturer’s instructions (Promega, Madison, WI, USA).

### TUNEL assay

TUNEL assay was used to measure the cell death of N2A cells after transfection of EV, WT-CPE and TC-CPE in the presence of H_2_O_2_. TUNEL assay was performed as described previously[11].

### Statistical analysis

All statistical analyses were performed by one-way analysis of variance (ANOVA) followed by Tukey's post-hoc multiple comparisons tests or student’s t-test. P value below 0.05 was considered statistical significance.

## Results

### Bioinformatic search identifies a single nucleotide polymorphism in the hCPE gene

**Fig 1:**
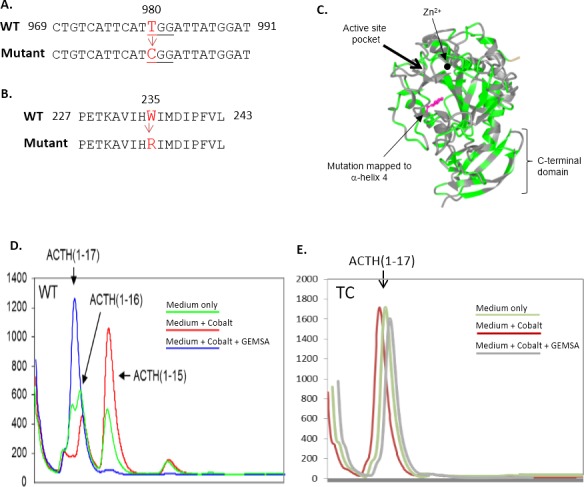
CPE mutation and the effect on enzymatic activity. **(A)**: Nucleotide and (**B)**: amino acid alignment of *WT-hCPE* and a human *CPE* mutant identified from an EST database. The mutant was named as *TC-CPE* due to the T to C mutation of *hCPE* gene at nucleotide 980 of exon 4. **C:** X-ray crystal structure of the catalytic domain of duck carboxypeptidase D (CPD, Protein Data Bank ID #1qmu), a homologous protein to CPE. The TC mutation occurs in the a-helix 4of CPD (see arrow). Condition medium from **(D)**WT-CPE and **(E)** TC-CPE transfected COS-7 cells were collected. The enzymatic activity of CPE from the conditioned medium was analyzed by HPLC. Condition medium from untransfected COS-7 cells were also analyzed and found to have no CPE activity (data not shown).

A single nucleotide polymorphism database (dbSNP) Blast analysis identified a T to C mutation at nucleotide 980 in the human *CPE* gene (Fig 1A), henceforth referred to as the *TC-CPE* mutant. This point mutation introduces a novel tryptophan to arginine (W235R) substitution in the catalytic domain of hCPE (Fig 1B and 1C). This mutation was found in 12.5% of the AGI_ASP population of patients that were in their 20s when their blood was analyzed. The AGI_ASP population is made up of 40 African-Americans and Caucasians.

### TC-CPE protein is enzymatically inactive

As the mutation of TC-CPE is in the catalytic domain, we first explored whether the TC-CPE mutant has enzymatic activity. COS-7 cells were used to transfect EV, WT-CPE and TC-CPE, as no endogenous CPE is expressed in this cell line. HPLC analysis of the products, generated by WT CPE, shows ACTH(1–16), ACTH(1–15), and ACTH(1–14) (Fig 1D, green curve). hCPE is a metallo-protease which activity is stimulated bycobalt. Fig 1D (red curve) shows an increase in ACTH(1–15) in the presence of cobalt (compare red with green curve) demonstrating a cobalt induced carboxypeptidase E activity. The lack of processed products with GEMSA treatment (blue curve), a known specific inhibitorof CPE, demonstrated that the activity is from CPE. Fig 1E shows the enzymatic activity of TC-CPE. The red curve represents the substrate and products after incubation of substrate with TC-CPE. Only one peak, the ACTH(1–17) substrate was present. This indicates that the TC-CPE does not have any enzymatic activity. Additionally, GEMSA (grey curve) and cobalt (green curve) has no effect on the TC-CPE.

### TC-CPE shows poor survival and neuroprotective effect compared with WT-CPE in N2A cells

**Fig 2:**
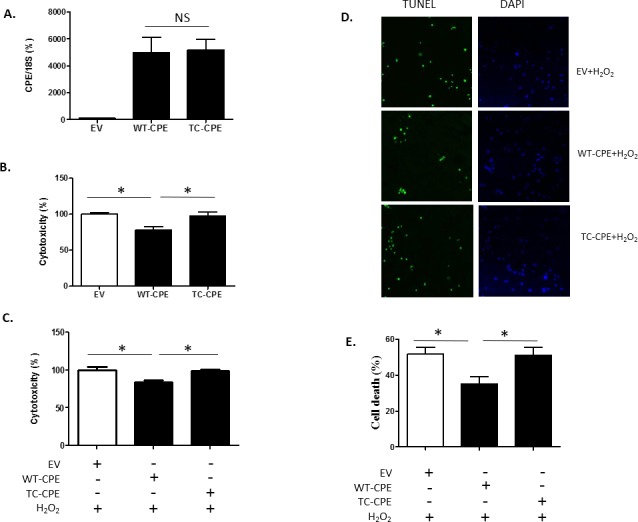
Over-expression of TC-CPE on neuroprotection. **(A)** N2A cells were transfected with EV, WT-CPE or TC-CPE for 24 h. The qRT-PCR showed no significant differences in transcript expression between WT-CPE and TC-CPE. t-test, p>0.05, n = 4. **(B)** N2A cells were transfected with EV, WT-CPE or TC-CPE for 72 h. The LDH release assay shows significantly reduced LDH released from cells transfected with WT-CPE compared to the EV group, while cells expressing TC-CPE had no significant effect. One-way ANOVA analysis followed by Tukey’s multiple comparison test: F_(2, 27)_ = 6.93, p<0.01,n = 10. **(C)** N2A cells were transfected with EV, WT-CPE or TC-CPE for 24 h, and then 100 μM H_2_O_2_ was added for 48h. The results from the LDH release assay showed that cells expressing WT-CPE were protected against the oxidative stress, while cells expressing TC-CPE werenot. One-way ANOVA analysis followed by Tukey’s multiple comparison test: F_(2, 12)_ = 8.99, p<0.01, n = 5. (D) TUNEL staining of N2A cells transfected with EV, WT-CPE or TC-CPE for 24 h, and then 100 μM H_2_O_2_ was added for 48 h. **(E)** The bar graph represents the quantification of the dead cells as a % of the total number of cells determined by the DAPI staining. One-way ANOVA analysis followed by Tukey’s multiple comparison test: F_(2, 9)_ = 5.581, p <0.05, n = 4.

Since CPE has been shown to be a survival and neuroprotective factor[10,11,20], we tested whether the T to C mutation at 980 sitein *CPE* gene would affect these properties. We first analyzed the transcript expression of WT-CPE and TC-CPE. N2A cells were transfected with EV, WT-CPE and TC-CPE for 24 h, and then qRT-PCR was used to detect the mRNA expression in the cells. No significant differences were found in the transcript expression between WT-CPE and TC-CPE (Fig 2A), suggesting TC-CPE had the same transfection efficiency as WT-CPE. Next, N2A cells were transfected with EV, WT-CPE or TC-CPE for 72 h, and then the LDH release assay was used to measure the cytotoxicity. As shown in Fig 2B, transfection of WT-CPE significantly reduced the cytotoxicity compared with EV transfected cells as analyzed by LDH release assay. Moreover, when challenged with H_2_O_2_, transfection of WT-CPE protected the N2A cells against the oxidative stress induced cytotoxicity, while TC-CPE had no effect (Fig 2C). Results from the TUNEL assay further confirmed that TC-CPE had no protective effect, in contrast to WT-CPE (Fig 2D and 2E). Furthermore, TC-CPE mutant did not cause enhanced cell death compared to cells transfected with EV (Fig 2B–2E). These results suggest that this mutation of CPE could not act in the same capacity as WT CPE with respect to neuroprotection.

### Expression and secretion of TC-CPE in N2A cells

**Fig 3:**
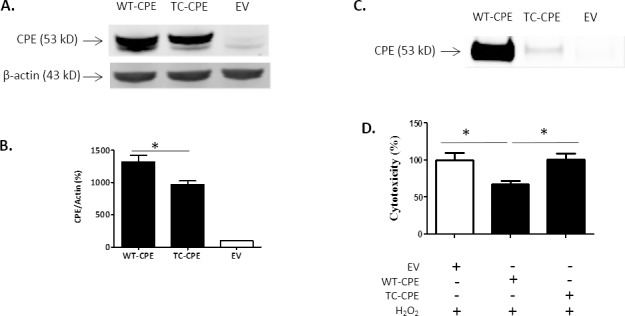
Expression and secretion of TC-CPE in N2A cells. **(A)** Representative Western blot analysis of CPE expression after EV, WT-CPE or TC-CPE transfection for 24 h in N2A cells. **(B)** Bar graphs show the quantification of CPE expression normalized to actin. One-way ANOVA (F_(2,6)_ = 85.04, p<0.001, n = 3) followed by Tukey’s multiple comparison test. **(C)** Representative Western blot analysis of secreted CPE after transfection of EV, WT-CPE or TC-CPE for 24 h in N2A cells. (D) N2A cells were treated with EV, WT-CPE or TC-CPE condition medium for 48 h in the presence of 100 μM H_2_O_2_. LDH release assay demonstrated that WT-CPE condition medium protected against oxidative stress, but not TC-CPE condition medium.One-way ANOVA analysis followed by Tukey’s multiple comparison test: F_(2,12)_ = 5.842, p <0.05, n = 5.

We next explored the potential mechanism underlying the loss of function of TC-CPE. N2A cells were transfected with EV, WT-CPE or TC-CPE for 24 h, and then the condition medium and cell lysates were collected after the treatments. Results from Western blot analysis showed that TC-CPE was present in lesser amount in the cells compared to WT-CPE (Fig 3A and 3B). Furthermore, TC-CPE was poorly secreted into the medium compared to WT-CPE (Fig 3C), suggesting that the correct trafficking of CPE through the secretory pathway was greatly compromised once it bears the T to C mutation in the catalytic domain, and the mutant CPE may be trapped in endoplasmic reticulum (ER). In addition, we collected condition medium from N2A cells transfected with EV, WT-CPE or TC-CPE for 24 h, and found that WT-CPE condition medium protected against oxidative stress induced toxicity in N2A cells, whereas TC-CPE condition medium did not have protective effect (Fig 3D). There results suggest that the loss of neuroprotective effect of TC-CPE is likely due to the diminished secretion from cells.

### TC-CPE "hijacks" WT-CPE in N2A cells

**Fig 4:**
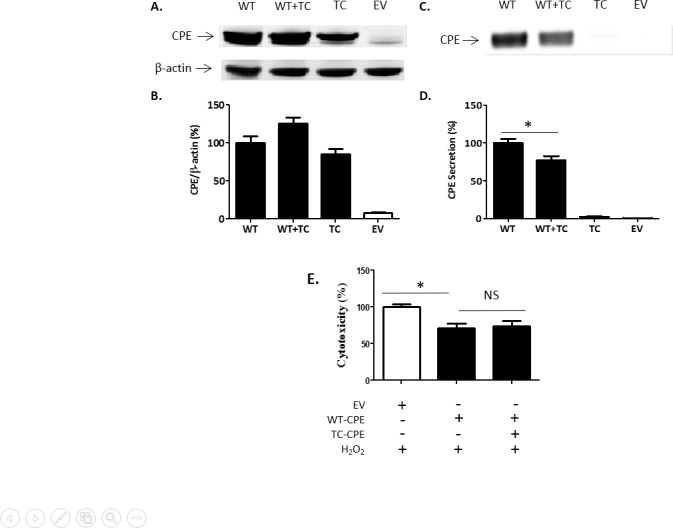
TC-CPE “hijacks” WT CPE in N2A cells. **(A)** Representative Western blot analysis of CPE expression from cell lysates after various treatments for 24 h in N2A cells. **(B)** Bar graphs show the quantification of CPE expression from cell lysates normalized to actin. **(C)** Representative Western blot analysis of secreted CPE after various treatments for 24 h in N2A cells. **(D)** Bar graphs show the quantification of secreted CPE levels. For CPE secretion, one-way ANOVA (F_(3,8)_ = 164.9, p<0.001, n = 3) followed by Tukey’s multiple comparison test. **(E)** N2A cells were transfected with EV, WT-CPE or WT-CPE +TC-CPE for 24 h, and then 100 μM H_2_O_2_ was added for 48 h. LDH release assay indicated that the neuroprotection of WT-CPE against oxidative stress was not affected by co-transfection with TC-CPE in N2A cells. One-way ANOVA analysis followed by Tukey’s multiple comparison test: F_(2,12)_ = 7.139, p < 0.05, n = 5.

We next investigated whether TC-CPE can influence the trafficking and secretion of WT-CPE in the cells. TC-CPE was co-transfected with WT-CPE in the N2A cells for 24 h and then the condition medium was collected for Western blot analysis. The results showed that the secretion of WT-CPE was significantly reduced when TC-CPE was co-expressed with WT-CPE, compared to cells transfected with WT-CPE alone (Fig 4C and 4D). Co-transfection of WT-CPE and TC-CPE did not block the WT-CPE mediated neuroprotection against oxidative stress in N2A cells (Fig 4E). This is likely due to sufficient WT-CPE being secreted in the medium to mediate the neuroprotection (Fig 4C and 4D).

### TC-CPE causes ER stress in N2A cells

**Fig 5:**
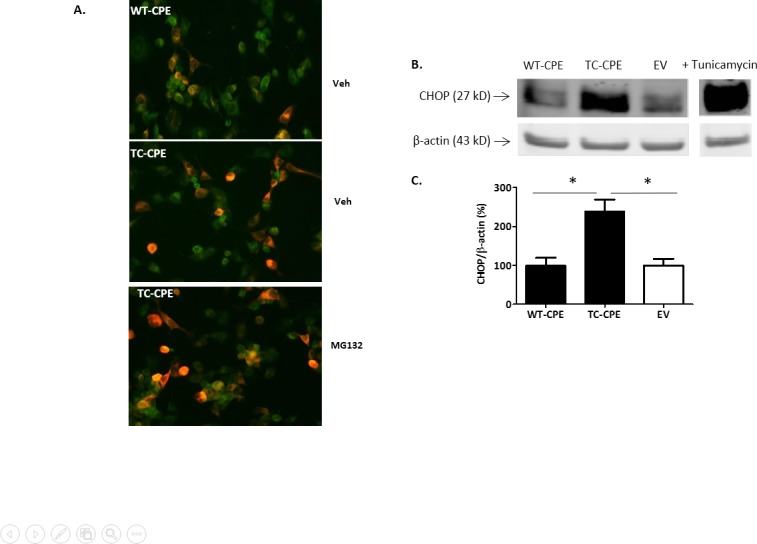
Over-expression of TC-CPE up-regulates CHOP levels. **(A)** Immunofluorescence of N2A cells expressing WT-CPE and TC-QQ. Double immunostaining of CPE (green) with calnexin (red) in N2A cells. Note that TC-CPE is co-localized with calnexin (orange) in the perinuclear area and accumulated in the ERin N2A cells, and apparent enhancement of the accumulation of TC-CPE in the ER with MG132 treatment. **(B)** Representative Western blot analysis of CHOP expression after EV, WT-CPE or TC-CPE transfection for 24 h in N2A cells. 10 μM tunicamycin was added to N2A cell for 24 h as positive control. **(C)** Bar graphs show the quantification of CHOP expression normalized to actin. One-way ANOVA (F_(2,9)_ = 11.55, p<0.01, n = 4) followed by Tukey’s multiple comparison test.

We next tested whether TC-CPE was accumulated in ER. Double labeling of CPE and Calnexin (an ER marker) in N2A cells showed that TC-CPE was accumulated in ER compared to control cells over-expressing WT-CPE. Moreover, the accumulation of TC-CPE appears to be enhanced in cells treated with the proteasome inhibitor, MG-132 (Fig 5A). Trapping and accumulation of TC-CPE in the ER would be expected to induce ER stress as it is well known that the unfolded protein response (UPR) is a cellular stress response related to the ER. Indeed, data from Western blots showed that over-expression of TC-CPE for 24 h in N2A cells significantly increased the expression of CHOP (Fig 5B and 5C), an ER stress marker. In contrast, over-expression of WT-CPE for 24 h did not cause ER stress in the cells.

### Proteasome degradation pathway of accumulated TC-CPE

**Fig 6:**
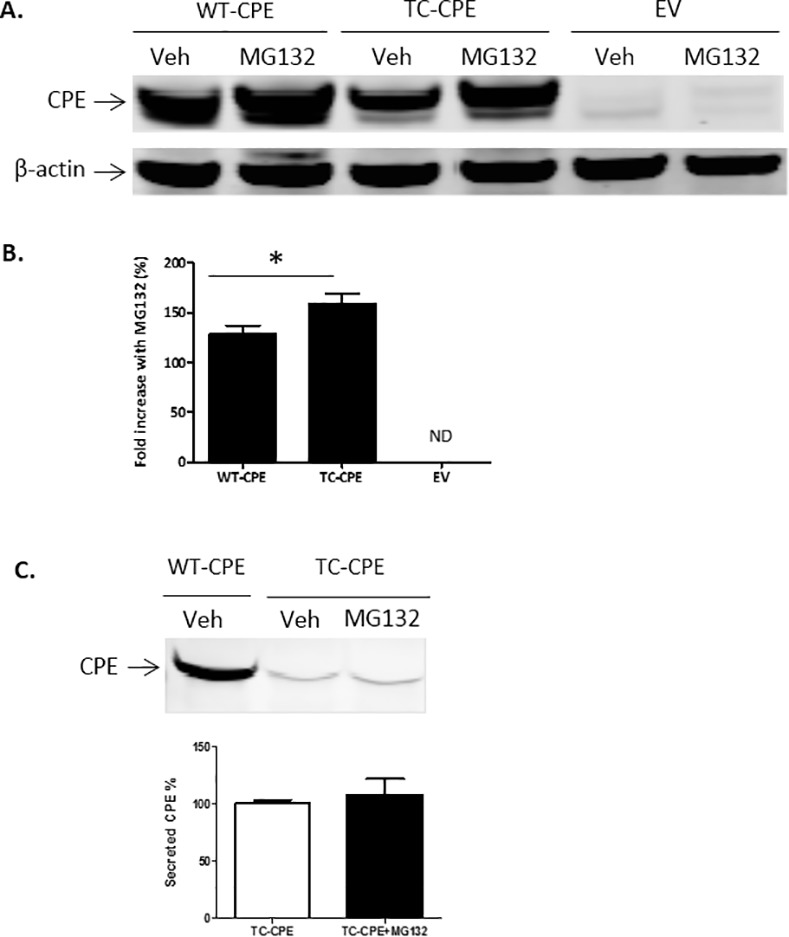
Proteasome degradation pathway of TC-CPE in N2A cells. (**A**) Representative Western blot analysis of CPE protein levels in N2A cells transfected with EV, WT-CPE, TC-CPE, and treated with MG132 or without (Veh). (**B**) Bar graphs show fold increase (%) CPE protein normalized to actin, in treated versus untreated cells transfected with EV, WT-CPE or TC-CPE. ND: Not detectable. One-way ANOVA (F_(2,15)_ = 14.16, p <0.001, n = 6) followed by Tukey’s multiple comparison test, *p<0.05 compared to EV. (**C**) Western blot analysis of secreted CPE protein levels in N2A cells transfected with WT-CPE or TC-CPE, in the presence or absence of MG132. Note that MG132 did not affect the secreted levels of TC-CPE protein in N2A cells. t-test, p > 0.05, n = 4.

Since we showed that CPE was trapped and accumulated in the ER, we hypothesized that the decreased expression of TC-CPE compared with WT-CPE is due to the activated UPR by misfolded TC-CPE which is then degraded through the proteasome system. Using the proteasome inhibitor, MG-132 we found that the inhibitor significantly increased the level of TC-CPE in the cells compared to WT-CPE group (Fig 6A and 6B), suggesting that the TC-CPE was targeted for degradation in the proteasome system. In addition, N2A cells treated with MG132 did not significantly influence the secretion levels of TC-CPE (Fig 6C).

## Discussion

The results obtained in this study indicate that a single nucleotide T980C mutation in the human *CPE* gene can cause loss of enzymatic activity of the CPE enzyme. We also showed that expression levels of this hCPE mutant were reduced compared to WT-CPE in N2A cells. In addition, the mutant was poorly secreted compared to WT-CPE. Our findings indicate that the mutant was synthesized and retained in the ER likely due to misfolding, and caused ER stress as evidenced by the increase in CHOP, a marker for ER stress-induced cell death. TC-CPE was eventually degraded through the proteasome pathway since the degradation was blocked by a proteasome inhibitor. This accounts for the diminished secretion of TC-CPE into the cell medium.

As CPE has been found to act as a trophic factor extracellularly to promote cell survival and neuroprotection[11], it is not surprising to find that over-expression of TC-CPE did not promote the survival of N2A cells with or without oxidative stress. This loss of function can be accounted for by the decreased secretion of the TC-CPE from cells. This is supported by our results showing that WT-CPE condition medium protected against oxidative stress in N2A cells, whereas TC-CPE condition medium did not have neuroprotective effect. The loss of function of the mutant also could be accounted for by the disruption of normal protein folding in the ER. Accumulation of unfolded or misfolded proteins in the ER would trigger an evolutional conserved response termed the unfolded protein response (UPR). The initial aim of the UPR is to reestablish normal ER function in response to changing environment. These adaptive mechanisms involve transcriptional programs that induce increase production of molecular chaperones involved in protein folding, and removing misfolded proteins. If the stress is prolonged, the goal of the UPR switches to commit the cell to a pathway of apoptosis[21,22]. One of the major pro-apoptotic proteins is CHOP which is uniquely responsive to ER stress[23]. We found that TC-CPE increased the production of CHOP, suggesting the increased ER stress in the presence of TC-CPE in the cells. TC-CPE induced ER stress was further supported by our results that TC-CPE were trapped and accumulated in the ER, and more apparent accumulation of TC-CPE was found in the ER after cells were treated with a proteasome inhibitor. As ER stress induced apoptosis has been implicated in various neurodegenerative diseases[24], the loss of function mutation in the *CPE* gene is likely to be involved in neurological disorders. In fact, mutation in the *CPE* gene has been found in the brain from a patient with Alzheimer’ disease[10]. Furthermore, a person who bears homozygosity for a truncating mutation of the *CPE* gene displayed intellectual disability[19]. The hypothesis that *CPE* mutation would contribute to neurological disorders is also supported by the animal studies showing that CPE-KO mice had the neuronal loss of the CA3 region of hippocampus, displayed memory deficits and depressive-like behaviors[12,13,20].

Due to the lack of enzymatic activity, this mutation in the *CPE* gene could result in or contribute towards diseases such as diabetes, obesity and infertility as were observed in the CPE knock mouse and mice bearing the Ser202Pro mutation lacking CPE [7,12,18]. Indeed, a recent study showed that a truncating mutation of the *CPE* gene found in a female from a consanguineous family, led to morbid obesity, intellectual disability, hypogonadotrophic hypogonadism and abnormal glucose homeostasis[19]. Additionally, a number of mutations in the human *CPE* gene have been reported and some are associated with endocrine problems. A C to T mutation in the *CPE* gene at nucleotide847 which caused a reduction of enzymatic function was found in a cohort of Jewish patients that have type 2 diabetes and it was associated with the early onset of diabetes [25]. In coronary atherosclerosis studies, point mutations of the *CPE* gene were associated with the disease from Chinese patients [26,27]. Accordingly, the T to C mutation of the *CPE* gene identified in our study which compromises the enzymatic activity of CPE in the AGI_ASP population would likely cause endocrine disorders. Thus these people who bear the mutation especially homozygous mutation are at high risk of developing obesity and diabetes in their later life. Unfortunately, we don’t have the detailed information of these patients and it is unlikely these patients have been followed-up.

Although we have characterized the property of the *CPE* mutation on its biochemistry and cell biological effects, future studies should give rise to more information on this and other types of *CPE* mutations. One possibility would be to generate transgenic mice carrying the T to C mutation in the *CPE* gene to study the *in vivo* effect of this mutation. Additionally, this mutation should be screened in larger populations to evaluate whether it exists in the general population. If this were the case, restrospective and prospective studies would help to determine if this mutation is a risk factor for diseases such as diabetes, obesity or poor memory.
